# Supplementary material for: Rhythmic sampling of multiple decision alternatives in the human brain
Source: Nat Commun. 2026 Feb 11;17:1587. doi: 10.1038/s41467-026-69379-z (PMC12901030; doi:10.1038/s41467-026-69379-z)
Supplement: Supplementary file 2 — Reporting Summary [file 41467_2026_69379_MOESM2_ESM.pdf]

nature portfolio

Corresponding author(s): Marcus Siems

Last updated by author(s): Nov 17, 2025

Reporting Summary

Nature Portfolio wishes to improve the reproducibility of the work that we publish. This form provides structure for consistency and transparency in reporting. For further information on Nature Portfolio policies, see our Editorial Policies and the Editorial Policy Checklist.

Statistics

For all statistical analyses, confirm that the following items are present in the figure legend, table legend, main text, or Methods section.

n/a	Confirmed	
☐	☒	The exact sample size (*n*) for each experimental group/condition, given as a discrete number and unit of measurement
☐	☒	A statement on whether measurements were taken from distinct samples or whether the same sample was measured repeatedly
☐	☒	The statistical test(s) used AND whether they are one- or two-sided<br>*Only common tests should be described solely by name; describe more complex techniques in the Methods section.*
☐	☒	A description of all covariates tested
☐	☒	A description of any assumptions or corrections, such as tests of normality and adjustment for multiple comparisons
☐	☒	A full description of the statistical parameters including central tendency (e.g. means) or other basic estimates (e.g. regression coefficient) AND variation (e.g. standard deviation) or associated estimates of uncertainty (e.g. confidence intervals)
☐	☒	For null hypothesis testing, the test statistic (e.g. *F*, *t*, *r*) with confidence intervals, effect sizes, degrees of freedom and *P* value noted<br>*Give P values as exact values whenever suitable.*
☒	☐	For Bayesian analysis, information on the choice of priors and Markov chain Monte Carlo settings
☒	☐	For hierarchical and complex designs, identification of the appropriate level for tests and full reporting of outcomes
☐	☒	Estimates of effect sizes (e.g. Cohen's *d*, Pearson's *r*), indicating how they were calculated

Our web collection on statistics for biologists contains articles on many of the points above.

Software and code

Policy information about availability of computer code

Data collection	Matlab2020a (Psychtoolbox3)
Data analysis	Matlab2020a (FieldTrip Version 20201009 & custom code)

For manuscripts utilizing custom algorithms or software that are central to the research but not yet described in published literature, software must be made available to editors and reviewers. We strongly encourage code deposition in a community repository (e.g. GitHub). See the Nature Portfolio guidelines for submitting code & software for further information.

Data

Policy information about availability of data

All manuscripts must include a data availability statement. This statement should provide the following information, where applicable:

- Accession codes, unique identifiers, or web links for publicly available datasets
- A description of any restrictions on data availability
- For clinical datasets or third party data, please ensure that the statement adheres to our policy

The processed data generated in this study has been deposited in the osf.io database under accession code [https://osf.io/tf6bw/].

April 2023

Research involving human participants, their data, or biological material

Policy information about studies with human participants or human data. See also policy information about sex, gender (identity/presentation), and sexual orientation and race, ethnicity and racism.

Reporting on sex and gender	We collected data from 20 healthy, right-handed participants and collected the sex (9 female, 11 male participants).
Reporting on race, ethnicity, or other socially relevant groupings	Race, ethnicity, sexual orientation, religious believes, socioeconomic status or occupation were not recorded in the current study.
Population characteristics	Participants reported their age and biological sex. Further, we excluded participants with a known neurological or psychiatric history (self-report) and left-handed participants. The mean age was 28.05y with a standard deviation of 4.60y. We refrain from conducting post hoc sex- or age-based analysis due to a low sample size within the groups. However, further research is needed to explicate rhythmic attentional sampling in other (sub-)populations (e.g., of different age and for varying socioeconomic status). We further cannot exclude a self-selection bias (see below "Recruitment').
Recruitment	The participants were recruited from a central pool of volunteers registered with the University Medical Center Hamburg Eppendorf (UKE) through anonymous emails and through flyers at lectures at the UKE.
Ethics oversight	Ethik-Kommision der Aerztekammer Hamburg

Note that full information on the approval of the study protocol must also be provided in the manuscript.

Field-specific reporting

Please select the one below that is the best fit for your research. If you are not sure, read the appropriate sections before making your selection.

☒ Life sciences ☐ Behavioural & social sciences ☐ Ecological, evolutionary & environmental sciences

For a reference copy of the document with all sections, see nature.com/documents/nr-reporting-summary-flat.pdf

Life sciences study design

All studies must disclose on these points even when the disclosure is negative.

Sample size	The sample size was derived after considering the past literature on the relevant behavioural effects (compare for example Dugue, Marque & VanRullen, JCogNeuro (2015), Berkowitsch et al., JExpPsychol (2013), Louie, Khaw & Glimcher PNAS (2013), Gluth et al., NatHumBeh (2020)). We were opting to attain as large sample sizes as possible (given time and resource constraints) and to perform multiple sessions (2 for the reported results) in order to maximize the statistical power in our experiments. For a conclusive comparisons among population covariates (for example sex or age differences) the sample is size too small.
Data exclusions	Being younger than 18 years old or older than 65 years old; Previously or currently diagnosed with neurological or psychiatric disorders; Family history of epilepsy; Pregnancy; Claustrophobia; Cardiovascular disease; Using illicit drugs; Rejection of information about unexpected incidental findings in structural MRI/ MEG; Pacemaker or other implanted biomedical devices (e.g. insulin pump, aneurysm clip, electrical stimulator for nerves/brain, intra-cardiac lines), or other foreign objects implanted in the body; Other implants, or metal objects in and around the body that are not MRI/MEG compatible; Impaired temperature sensation and / or increased sensitivity to heat.
Replication	The central results of the presented manuscript are likely general characteristics of the central nervous system and are expected to be reproducible. We collected data from an additional cohort of 24 participants performing a compatible task (3-alternative protracted decision-making) and could reproduce attentional rhythmicity between 8-12Hz. No analyses within that task contradict the here reported findings. These results are part of a distinct project and will be made publicly available as part of an independent publication.
Randomization	Experimental variables (contrasts, grating orientations, task framing) were randomized within subjects over trials. The foot response to the top-option was randomized between subjects.
Blinding	We could not blind the between-subject factor (foot-response) because we only had access to one foot-pedal and needed to instruct the participants on the choice-response mapping.

Reporting for specific materials, systems and methods

We require information from authors about some types of materials, experimental systems and methods used in many studies. Here, indicate whether each material, system or method listed is relevant to your study. If you are not sure if a list item applies to your research, read the appropriate section before selecting a response.

Materials & experimental systems

n/a	Involved in the study
☒	☐ Antibodies
☒	☐ Eukaryotic cell lines
☒	☐ Palaeontology and archaeology
☒	☐ Animals and other organisms
☒	☐ Clinical data
☒	☐ Dual use research of concern
☒	☐ Plants

Methods

n/a	Involved in the study
☒	☐ ChIP-seq
☒	☐ Flow cytometry
☒	☐ MRI-based neuroimaging

Plants

Seed stocks	*Report on the source of all seed stocks or other plant material used. If applicable, state the seed stock centre and catalogue number. If plant specimens were collected from the field, describe the collection location, date and sampling procedures.*
Novel plant genotypes	*Describe the methods by which all novel plant genotypes were produced. This includes those generated by transgenic approaches, gene editing, chemical/radiation-based mutagenesis and hybridization. For transgenic lines, describe the transformation method, the number of independent lines analyzed and the generation upon which experiments were performed. For gene-edited lines, describe the editor used, the endogenous sequence targeted for editing, the targeting guide RNA sequence (if applicable) and how the editor was applied.*
Authentication	*Describe any authentication procedures for each seed stock used or novel genotype generated. Describe any experiments used to assess the effect of a mutation and, where applicable, how potential secondary effects (e.g. second site T-DNA insertions, mosiacism, off-target gene editing) were examined.*
